# SARS‐CoV‐2, an evolutionary perspective of interaction with human ACE2 reveals undiscovered amino acids necessary for complex stability

**DOI:** 10.1111/eva.12980

**Published:** 2020-05-07

**Authors:** Vinicio Armijos‐Jaramillo, Justin Yeager, Claire Muslin, Yunierkis Perez‐Castillo

**Affiliations:** ^1^ Grupo de Bio‐Quimioinformática Carrera de Ingeniería en Biotecnología Facultad de Ingeniería y Ciencias Agropecuarias Universidad de Las Américas Quito Ecuador; ^2^ Biodiversidad Medio Ambiente y Salud (BIOMAS) Dirección General de Investigación Universidad de Las Américas Quito Ecuador; ^3^ One Health Research Group Faculty of Health Sciences Universidad de Las Américas Quito Ecuador; ^4^ Grupo de Bio‐Quimioinformática y Escuela de Ciencias Físicas y Matemáticas Universidad de Las Américas Quito Ecuador

**Keywords:** ACE2, coronavirus, molecular dynamics, positive selection, purifying selection, SARS‐CoV‐2, spike protein

## Abstract

The emergence of SARS‐CoV‐2 has resulted in nearly 1,280,000 infections and 73,000 deaths globally so far. This novel virus acquired the ability to infect human cells using the SARS‐CoV cell receptor hACE2. Because of this, it is essential to improve our understanding of the evolutionary dynamics surrounding the SARS‐CoV‐2 hACE2 interaction. One way theory predicts selection pressures should shape viral evolution is to enhance binding with host cells. We first assessed evolutionary dynamics in select betacoronavirus spike protein genes to predict whether these genomic regions are under directional or purifying selection between divergent viral lineages, at various scales of relatedness. With this analysis, we determine a region inside the receptor‐binding domain with putative sites under positive selection interspersed among highly conserved sites, which are implicated in structural stability of the viral spike protein and its union with human receptor ACE2. Next, to gain further insights into factors associated with recognition of the human host receptor, we performed modeling studies of five different betacoronaviruses and their potential binding to hACE2. Modeling results indicate that interfering with the salt bridges at hot spot 353 could be an effective strategy for inhibiting binding, and hence for the prevention of SARS‐CoV‐2 infections. We also propose that a glycine residue at the receptor‐binding domain of the spike glycoprotein can have a critical role in permitting bat SARS‐related coronaviruses to infect human cells.

## 1 INTRODUCTION

The recent emergence of the novel SARS coronavirus 2 (SARS‐CoV‐2) marked the third introduction of a highly pathogenic coronavirus into the human population in the twenty‐first century, following the severe acute respiratory syndrome coronavirus (SARS‐CoV) and the Middle East respiratory syndrome coronavirus (MERS‐CoV). The first SARS‐CoV emerged in November 2002 in the Guangdong province of China and spread globally during 2002–2003, infecting more than 8,000 people and causing 774 deaths (Drosten et al., 2003; WHO, 2004). MERS‐CoV was the second emergence and was first detected in Saudi Arabia in 2012 and resulted in nearly 2,500 human infections and 858 deaths in 27 countries (Fehr, Channappanavar, & Perlman, 2017; Zaki, Boheemen, Bestebroer, Osterhaus, & Fouchier, 2012). In December 2019, SARS‐CoV‐2, a previously unknown coronavirus capable of infecting humans was discovered in the Chinese city of Wuhan, in the Hubei Province (Huang et al., 2020; Zhu et al., 2020). SARS‐CoV‐2 is associated with an ongoing pandemic of atypical pneumonia, now termed coronavirus disease 19 (Covid‐2019) that has affected over 1,280,000 people with 72,614 fatalities as of April 7, 2020 (WHO, 2020). Both SARS‐CoV and MERS‐CoV are thought to have originated in colonies of bats, eventually transmitted to humans, putatively facilitated by intermediate hosts such as palm civets and dromedary camels, respectively (Cui, Li, & Shi, 2019). The genome of SARS‐CoV‐2 shares about 80% nucleotide identity with that of SARS‐CoV and is 96% identical to the bat coronavirus BatCoV RaTG13 genome, reinforcing the probable bat origin of the virus (Zhou et al., 2020). However, better assessing the evolutionary dynamics of SARS‐CoV‐2 is an active research priority worldwide.

SARS‐CoV, MERS‐CoV, and SARS‐CoV‐2 belong to the genus *Betacoronavirus* within the subfamily *Coronavirinae* of the family *Coronaviridae*. Members of this family are enveloped viruses containing a single positive‐strand RNA genome of 27–32 kb in length, the largest known RNA virus genome. The coronavirus spherical virion consists of four structural proteins: the spike glycoprotein (S‐protein), the envelope protein, membrane protein, and nucleocapsid. The transmembrane trimeric S‐protein plays a critical role in virus entry into host cells (Gallagher & Buchmeier, 2001; Tortorici & Veesler, 2019). It comprises two functional subunits: S_1_ subunit, where the receptor‐binding domain (RBD) is found, is responsible for binding host cell surface receptors and S_2_ subunit mediates subsequent fusion between the viral and cellular membranes (Kirchdoerfer et al., 2016; Yuan et al., 2017). Both SARS‐CoV and SARS‐CoV‐2 interact directly with angiotensin‐converting enzyme 2 (ACE2) to enter host target cells (Hoffmann et al., 2020; Li et al., 2003; Walls et al., 2020; Yan et al., 2020). In the case of SARS‐CoV, ACE2 binding was found to be a critical determinant for the range of hosts the virus can infect, and key amino acid residues in the RBD were identified to be essential for ACE2‐mediated SARS‐ CoV infection and adaptation to humans (Li et al., 2005, 2006).

Understanding the dynamics that permits a virus to shift hosts is of considerable interest and can further be an essential preliminary step toward facilitating the development of vaccines and the discovery of specific drug therapies. We employ a multidisciplinary approach to look for evidence of diversifying selection on the S‐protein gene and model the interactions between human ACE2 (hACE2) and the RBD of selected coronavirus strains, which ultimately afforded us novel insights detailing virus and host cell interactions. Given the rapid pace of discovery, we aim to add clarity to evolutionary dynamics of diseases strains by more precisely understand the dynamics at the S‐protein and its interaction with hACE2.

## 2 METHODS

### 2.1 Phylogenetic reconstruction and analysis testing for evidence of positive/purifying selection in the coronavirus S‐protein region

The most similar genomes to SARS‐CoV‐2 MN908947 were retrieved using BLASTp (Altschul et al., 1997) versus the NR database of GenBank (Table 1). Genomes were then aligned using MAUVE (Darling, Mau, Blattner, & Perna, 2004), and the S‐protein gene was trimmed. The extracted genomic sections were aligned using the translation align option of Geneious (Kearse et al., 2012) with a MAFFT plugin (Katoh & Standley, 2013). The phylogenetic reconstruction of S‐protein genes was performed with PhyML (Guindon et al., 2010), using a GTR + I + G model, using with 100 nonparametric bootstrap replicates. Both, the alignment and the tree were used as inputs for PAML Codeml (Yang, 2007). The presence of sites under positive selection was tested by the comparison of M2 model (which it allows for a proportion of positive, neutral, and negative selection sites in the alignment) versus the M1 model (it which only allows a proportion of neutral and negative selection sites in the alignment) and M8 (ω follows a beta distribution plus a proportion of sites with ω > 1) versus M7 (ω follows a beta distribution) models using the ETE toolkit 3.0 (Huerta‐Cepas, Dopazo, & Gabaldón, 2010). The presence of tree nodes under positive selection was obtained with the free branch model and then tested by the comparison of branch free (different ω for each selected branches) versus M0 (negative selection for all sites and branches‐null model) and branch free versus branch neutral (ω = 1 for selected branches) models. The presence of sites with positive selection under specific branches of the tree was tested with bsA (proportion of sites with positive selection in a specific branch of the tree) versus bsA1 (proportion of sites with neutral and purifying selection in a specific branch of the tree) models. Likelihood ratio test (LRT) was performed (*p* ≤ .05) to compare the hypothesis contrasted by each model.

**TABLE 1:** List of coronavirus isolates used for positive selection analysis (closer dataset)

Accession number (NCBI)	Description
AY390556	SARS coronavirus GZ02
DQ071615	Bat SARS‐like coronavirus Rp3
DQ084199	Bat SARS‐like coronavirus HKU3‐2
DQ412043	Bat SARS‐like coronavirus Rm1
FJ211859	Recombinant coronavirus clone Bat SARS‐CoV
FJ211860	Recombinant coronavirus clone Bat‐SRBD
KC881006	Bat SARS‐like coronavirus Rs3367
KF569996	Rhinolophus affinis coronavirus isolate LYRa11
KJ473814	BtRs‐BetaCoV/HuB2013
KT444582	SARS‐like coronavirus WIV16
KY417142	Bat SARS‐like coronavirus isolate As6526
KY417144	Bat SARS‐like coronavirus isolate Rs4084
KY417146	Bat SARS‐like coronavirus isolate Rs4231
KY417148	Bat SARS‐like coronavirus isolate Rs4247
MG772933	Bat SARS‐like coronavirus isolate bat‐SL‐CoVZC45
MG772934	Bat SARS‐like coronavirus isolate bat‐SL‐CoVZXC21
MK211375	Coronavirus BtRs‐BetaCoV/YN2018A
MK211378	Coronavirus BtRs‐BetaCoV/YN2018D
MN996532	Bat coronavirus RaTG13
MT084071	Pangolin coronavirus isolate MP789
MN908947	Wuhan seafood market pneumonia virus isolate Wuhan‐Hu‐1 (SARS‐CoV‐2)

We used the set of programs available in HyPhy (Kosakovsky Pond et al., 2020), Fast Unconstrained Bayesian AppRoximation (FUBAR) to detect overall sites under positive selection, and Fixed Effects Likelihood (FEL) to detect specific sites under positive selection in specific branches. We used Mixed Effects Model of Evolution (MEME) to detect episodic positive/diversifying selection and adaptive Branch Site REL (aBSREL) to detect branches in the tree under positive selection. The web server Datamonkey (Weaver et al., 2018) was used to perform the HyPhy analyses.

Finally, TreeSAAP 3.2 (Woolley, Johnson, Smith, Crandall, & McClellan, 2003) was used to detect sites under adaptation (in terms of physicochemical properties). The same alignment and tree described above were used for this analysis. All these experiments were performed again using the S‐protein genes of a shorter list of accessions and more distantly related (broad dataset) to SARS‐COV‐2 (AY304488, AY395003, DQ412043, FJ882957, KY417144, MG772933, MG772934, MN908947, NC_004718) to test the reproducibility of the predicted branches and sites under positive selection.

### 2.2 Assessing molecular structure and host‐specific interactions

The crystal structure of the SARS‐CoV S‐protein RBD in complex with hACE2 was retrieved from the Protein Data Bank (code 2AJF) (Berman et al., 2000). Homology models were constructed using this structure as template for the RBDs of SARS‐CoV‐2 (SARS2, GeneBank ID MN908947), the Bat SARS‐like coronavirus isolate Rm1 (Rm1, GeneBank ID DQ412043), and the Bat SARS‐like coronavirus isolate Rs4231 (Rs4231, GeneBank ID KY417146). One additional homology model for the G496D mutant of the SARS‐CoV‐2 RBD (SARS2‐MUT) was constructed. Homology models were built with Modeller v. 9 (Webb & Sali, 2016) using its UCSF Chimera interface (Pettersen et al., 2004). Five models were constructed for each target sequence and the one with the lowest Discrete Optimized Protein Energy (DOPE) score was selected for the final model.

All nonamino acidic residues were removed from the SARS‐CoV RBD‐hACE2 complex to obtain a clean complex. The homology models of the SARS2, Rm1, Rs4231 RBDs, and SARS2‐MUT were superimposed into the SARS‐CoV RBD to obtain their initial complexes with hACE2. These complexes were then subject to molecular dynamics (MD) simulations and estimation of their free energies of binding using Amber 18 (Case et al., 2018). For the later, ACE2 was considered as the receptor and the RBDs as ligands. The protocol described below was employed for all complexes and otherwise noted default software parameters were employed.

Systems preparation was performed with the tleap program of the Amber 18 suite. Each complex was enclosed in a truncated octahedron box extending 10 Å from any atom. Next, the boxes were solvated with TIP3P water molecules and Na+ ions were added to neutralize the excess charge. Systems were minimized in two steps, the first of which consisted in 500 steps of the steepest descent algorithm followed by 500 cycles of conjugate gradient with protein atoms restrained using a force constant of 500 kcal/mol.Å^2^. The PME method with a cutoff of 12 Å was used to treat long‐range electrostatic interactions. During the second minimization step, the PME cutoff was set to 10 Å and it proceeded for 1,500 steps of the steepest descent algorithm followed by 1,000 cycles of conjugate gradient with no restrains. The same PME cutoff of 10 Å was used in all simulation steps from here on. Both minimization stages were performed at constant volume.

The minimized systems were heated from 0 to 300 K at constant volume constraining all protein atoms with a force constant of 10 kcal/mol.Å^2^. The SHAKE algorithm was used to constrain all bonds involving hydrogens and their interactions were omitted from this step on. Heating took place for 10,000 steps, with a time step of 2 fs and a Langevin thermostat with a collision frequency of 1.0 ps^−1^ was employed. All subsequent MD steps utilized the same thermostat settings. Afterward, the systems were equilibrated for 100 ps at a constant temperature of 300 K and a constant pressure of 1 bar. Pressure was controlled with isotropic position scaling with a relaxation time of 2 ps. The equilibrated systems were used as input for 10 ns length production MD simulations.

The free energies of binding were computed under the MM‐PBSA approach implemented in AmberTools 18 (Case et al., 2018). A total of 100 MD snapshots were evenly selected, one every 50 ps, from the last 5 ns of the production run for MM‐PBSA calculations. The ionic strength was set to 100 mM and the solute dielectric factor was set to 4 for all systems.

## 3 RESULTS AND DISCUSSION

### 3.1 Selective constraints over spike protein in betacoronaviruses

In order to detect branches and sites under positive/negative selection, two datasets were explored. The first (“closer” dataset) harbors the most similar genomes to Wuhan‐Hu‐1 coronavirus (SARS‐CoV‐2) (MN908947). For this dataset, several genomes were excluded from the analysis because they showed minimal variation to other sequences. We used a preliminary phylogeny to select a representative isolate of each clade (Table 1) in order to exclude highly similar sequences. The second dataset (“broad” dataset) includes some accessions of the first dataset plus isolates less related to SARS‐CoV‐2, like SARS‐like coronavirus isolates from different countries (see methods). We compare the results of two dataset because the phylogenetic distance between orthologues in a given dataset has been demonstrated to alter the ability to detect selection in PAML and MEME (McBee, Rozmiarek, Meyerson, Rowley, & Sawyer, 2015).

**FIGURE 1:**
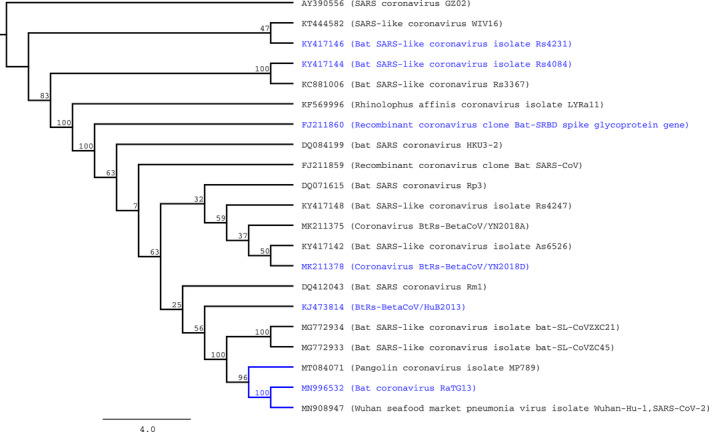
Phylogenetic tree of S‐protein genes of selected betacoronaviruses (“closer” dataset). Predicted nodes under episodic diversifying selection (according to predictions generated by aBSREL of HyPhy) were colored in blue. Numbers in the inner nodes represent the number of nonparametric bootstrap replicates

In both datasets, we observed evidence of purifying selection in the majority of nodes of the tree. Specifically, in the “closer” dataset, we identified 38 nodes with evidence of negative selection, and 4 under positive selection when free ratios model of CODEML model was applied. To confirm the four nodes under positive selection, we use LTR test for contrasting hypothesis using branch free, branch neutral, and M0 models of CODEML. Using these approximations, any node predicted by free ratios model with ω > 1 was significantly different to the purifying (ω < 1) or neutral (ω = 1) models. An equivalent analysis was performed using aBSREL of HyPhy, observing episodic diversifying selection in at least 8 of 41 nodes of the phylogenetic tree reconstructed with the “closer” dataset (Figure 1). Interestingly, one of the divisions detected with diversifying selection was the branch that contains SARS‐CoV‐2, Pangolin coronavirus isolate MP789 and bat coronavirus RaTG13 (called SARS‐CoV‐2 group) but not the specific branch that contains SARS‐CoV‐2.

MEME of HyPhy detected 40 sites in different branches with signal of episodic positive selection, but FEL did not identify sites under positive selection (with *p* < .1) in the SARS‐CoV‐2 when “closer” dataset was used. Depending on the focal dataset analyzed (“close” versus “broad”), FEL detected several sites under positive selection in SARS‐CoV‐2, demonstrating the influence of relatedness within the dataset in branch‐sites model predictions. FEL predicted the site F486 under positive selection in SARS‐CoV‐2 using the closer dataset without Pangolin coronavirus isolate MP789. It is interesting this result is robust despite the influence of the genetic diversity represented within dataset because site F486 is directly involved in hACE2‐RBD interaction (Shang et al., 2020), explaining at least in part strong selection at this site. Moreover, the branch‐site model bsA (positive selection) versus bsA1 (relaxation) of CODEML were compared to find evidence of sites under positive selection in branch of SARS‐CoV‐2 using the “closer” dataset. However, bsA model does not show significant differences when compared to bsA1 (*p* > .05) indicating selection cannot be confidently implicated; however, it was when other datasets like “broad” and “closer” without isolate MP789 were used. In summary, we do find evidence of sites under positive/episodic selection in branches of closely related strains of Wuhan‐Hu‐1 isolate coronavirus. However, there is not strong evidence of specific sites under positive selection in SARS‐CoV‐2 at least using the techniques implemented here. This result does not disregard the possibility of positive selection on sites in SARS‐CoV‐2; nonetheless, it highlights the limitation of these methods to identify, with precision, specific sites under positive selection in a precise sequence when different orthologue genes are compared. We further warn researchers need to be conservative with interpretations of studies utilizing these methodologies, given the equivocal results can be generated by datasets varying in genetic similarity.

To complement our analyses looking for evidence of selection among lineages, we specifically analyzed for patterns of selection across sites in the S‐protein genes and we used the sites models available in CODEML and HyPhY. Model M2 of CODEML detected 0.133% of sites under positive selection (ω > 1) and models M1 and M2 detected 85% of sites under purifying selection (ω < 1). Model M2 explains the significant data better (*p* = 7e‐4) than M1 model, that takes in account only sites with neutral and purifying selection. FUBAR of HyPhy also detected 1,070 sites under purifying selection and only 2 sites under positive selection (alignment size 1,284). A similar analysis performed by Benvenuto et al. (2020) with FUBAR detects 1,065 sites under purifying selection and sites 536 and 644 as under positive selection.

These results suggest by in large strong purifying selection is acting over the vast majority of the S‐protein gene, with a comparatively low proportion of sites under positive selection. Evidence suggesting large amounts of purifying selection strengthens similar findings previously reported in RNA viruses (Hughes & Hughes, 2007). These results are congruent with the analysis of Tang et al. (2020) performed with entire coronavirus genomes. However, the sites under positive selection reported differ from our results, and from those of Benvenuto et al. (2020). They found the sites 439, 483, and 439 (counting from the first amino acid of S‐protein), our FUBAR analysis found the sites 443 and 445, and FEL found the residue 486 as specific site under positive selection in SARS‐COV‐2. To resolve these ambiguities in positive selection sites, we calculate putative selection sites with CODEML (using Bayes Empirical Bayes from M2 and M8 models) and FUBAR with different datasets reflecting the addition of novel sequences to online repositories (“broad,” “closer,” “closer” without MN996532 and MT084071 and “closer” without MT084071) and we obtain different results.

It is becoming increasingly clear that predictions of positive selected sites are highly influenced simply by the genetic diversity of the individual sequences included in the analyzed datasets. Nonetheless, the majority of sites predicted to be under positive selection converge in the region between 439 and 508, a section of the RBD. Additionally, we used TreeSaap to detect important biochemical amino acid properties changes over regions and/or sites along betacoronavirus S‐protein. Using a sliding window size of 20 (increasing by 1), we detect that the region between 466 and 500 (using SARS‐COV‐2 S‐protein as a reference) have drastic amino acid changes for alpha‐helical tendencies. In addition, the section between residues 448 and 485 shows radical changes in amino acids implicated in the equilibrium constant (ionization of COOH). In the structural analysis we performed, the section between 472 and 486 forms a loop that is not present in certain S‐proteins of coronavirus isolated in bats. This loop extends the interaction area between RBD of S‐protein and human ACE2; in fact, the lack of this loop decreases the negative energy of interaction (increasing the binding) among these two molecules (see Table 2). These results, obtained from independent analyses, strongly highlight the importance of 439–508 region. Additionally, important hACE2‐binding residues in the RBD from SARS‐COV‐2 obtained from the crystallography and structure determination performed by Shang et al. (2020) are also present in the section we highlight here. We propose that this region is the most probable to contain the sites under positive selection due to predictions by our CODEML and FUBAR models. In that sense, we refer to this section as region under positive selection (RPS). It is important to additionally clarify that even inside the RPS we found at least 20 aa highly conserved between coronaviruses, several of them are predicted as sites under purifying selection. This shows that it is necessary to maintain conserved sites which are located around polymorphic sites, probably to maintain the protein structure and at the same time to have the ability to colonize more than one host.

**TABLE 2:** Estimated free energies of binding of the coronaviruses RBDs to ACE2. Energy values are expressed in kcal/mol

RBD	VDWAALS	EEL	EPB	ENPOLAR	EDISPER	ΔG TOTAL
SARS2	−89.08	−180.40	180.71	−64.30	129.87	−23.19
SARS	−88.94	−171.58	175.65	−64.10	133.81	−15.18
Rs4231	−80.93	−139.61	141.89	−56.96	119.89	−15.71
Rm1	−77.06	125.14	−101.69	−56.66	122.30	12.04
SARS2‐MUT	−83.88	−89.47	103.76	−59.49	127.43	−1.66

Interestingly, the RPS of the pangolin coronavirus isolate MP789 differs only in one amino acid with the homologous region of SARS‐CoV‐2, whereas in contrast, the bat coronavirus RaTG13 (the overall most similar isolate to SARS‐CoV‐2 sequenced at the moment) shows 17 differences in the very same region. Several explanations could be derived from this observation. The hypothesis of recombination inside pangolins, between a native coronavirus strain and a bat coronavirus (like RaTG13) is congruent with our observation. This scenario was recently proposed and discussed as the origin of SARS‐CoV‐2 (Lam et al., 2020; Wong, Cregeen, Ajami, & Petrosino, 2020; Xiao et al., 2020); however, other explanations are still plasusible. For instance, if the SARS‐CoV‐2, RaTG13 and pangolin coronavirus MP789 isolates are closely related as shown in the tree of the Figure 1, we are observing the ancestral sequence of RPS in human and pangolin coronaviruses, and a mutated version in bat virus. Elucidating the origin of SARS‐CoV‐2 is beyond the scope of this work; nevertheless, sequencing of new coronavirus isolates in the near future could resolve this question.

### 3.2 Structural analysis of spike protein and human ACE2

With a list of broader observations related to the role of selection across viral genomes, we aimed to specifically understand how these regions could affect virus/host interactions. To understand more in more detail the importance of RPS in the evolution of SARS‐COV‐2, we quantified the relative importance of this region in the interaction between the RBDs and hACE2. In that sense, MD simulations were run for five complexes (listed in methods). Simulations were initially performed for four RBDs corresponding to the SARS2, SARS, Rs4231 and Rm1 coronaviruses. As discussed below in this section, the G496D mutation is predicted to have a large negative influence in the stability of the RBD‐hACE2 complexes. To further clarify this influence, we added as fifth system a G496D SARS2 mutant RBD‐hACE2 complex in our studies.

In all cases, the systems were stable with root mean square deviations (RMSD) of their backbones between 1.11 and 3.30 Å relative to the initial complexes structures during the last 5 ns of the production run. We first investigated the network of contacts between the ligands (coronavirus RDB) with the receptor (hACE2). Overall, all complexes present a large number of contacts between the ligands and the receptor in at least 50% of the MD snapshots selected for MM‐PBSA calculations. Common interactions with T27, F28, K31, H34, Y41, K353, G354, D355, and R357 of the receptor are observed in all systems. The full networks of interactions between the selected betacoronaviruses and the hACE2 receptor are provided as Supporting Information.

**FIGURE 2:**
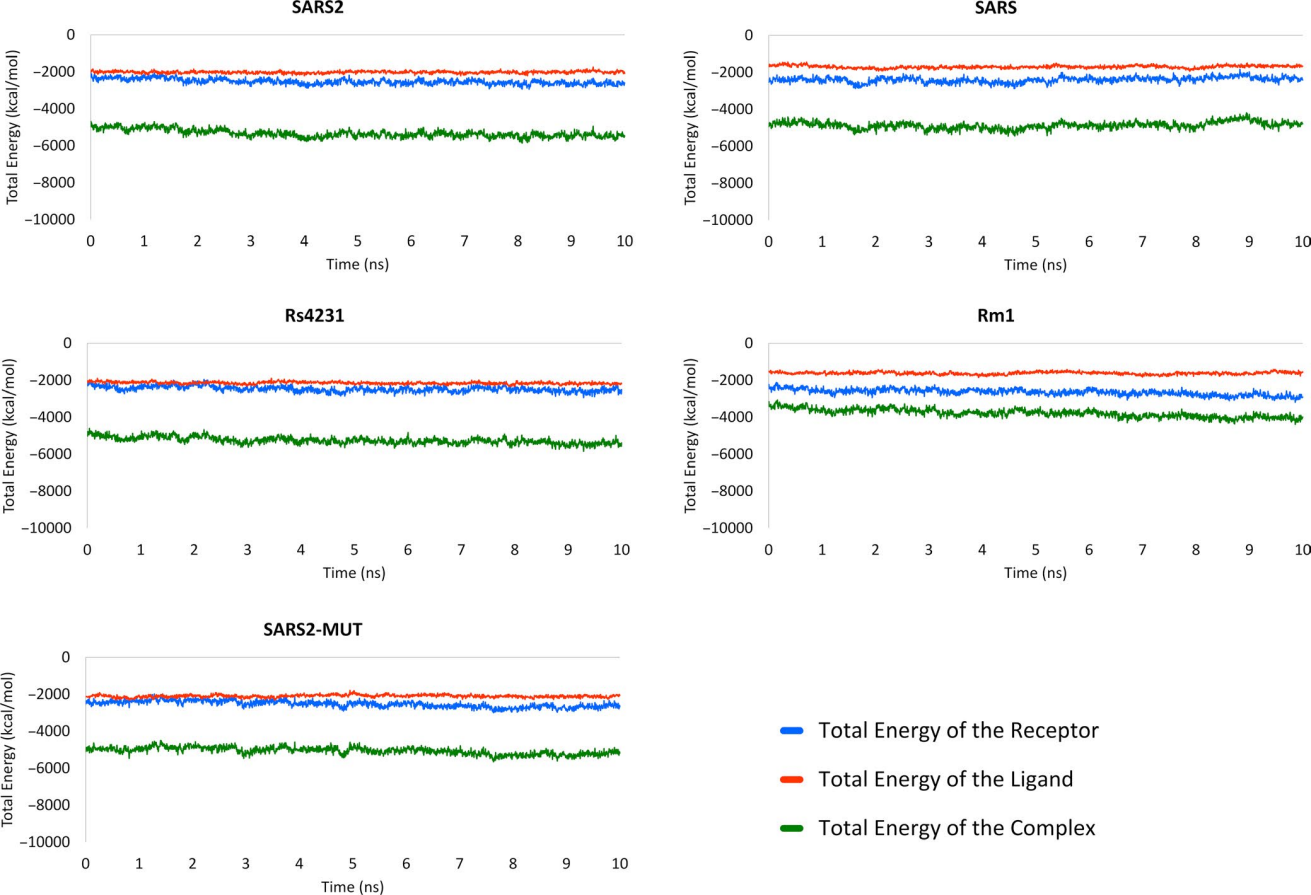
Plots of the total energy of the investigated RBDs‐hACE2 complexes along the 10 ns production MD simulations. Energy values are separately presented for ligands, receptors, and complexes

Next, we estimated the free energies of binding of the coronaviruses RBDs to hACE2 over 100 snapshots selected from the last 5 ns MD simulations as described in the Methods section. Prior to MM‐PBSA calculations, the energetic stabilization of the MD production runs was investigated. As seen from Figure 2, the total energy of the ligands, receptors, and complexes remain stable along the last 5 ns of MD simulations from which the MD snapshots for free energies of binding estimation are extracted. The larger energetic variability among any complex or its components is observed for the receptor of the SARS RDB‐hACE2 system and it does not exceed a coefficient of variation of 5.5%. In consequence, the energetic equilibrium of the simulated systems enables their use for MM‐PBSA calculations.

The predicted free energies of binding of the five simulated complexes are presented in Table 2. These calculations show that the SARS2, SARS, and Rs4231 viruses are predicted to favorably bind to the human hACE2 receptor, while the Rm1 and SARS2‐MUT variants present unfavorable free energies of binding. The fact that the bat coronavirus Rs4231, in addition to SARS and SARS2, presents favorable interaction with hACE2 is in accordance with the previous observation that it is able to infect human cells expressing this protein (Hu et al., 2017). To gain further insights into the contribution of the receptor and the RBDs to the binding process, we performed energy decomposition experiments over the same ensemble of MD snapshots used in MM‐PBSA calculations.

The contribution of each residue in the studied coronaviruses that interact with the hACE2 receptor are shown in Table 3. Rows are presented in such a way that each of them contains the residues occupying the same position in the viral RBDs structures as in the SAR2 RBD structure. From here on, residues numeration will take that of SARS2 as reference. In general, most RBDs residues show negative values of contribution to the free energies of binding to the human receptor. All studied RBDs, except that of the Rm1 coronavirus, have amino acids with large favorable contributions to the free energies of binding that directly interact with hACE2: K417 of SARS2 and SARS2‐MUT, R426 in SARS and R480 in Rs4231. On the other hand, the G476D mutation (D463 present in bat coronavirus strains) has a negative contribution to the binding of the RDB to hACE2. This site was predicted to be under purifying selection by FUBAR analyses, and is located within the RPS.

**TABLE 3:** Contribution of the residues in the RDB of the coronaviruses interacting with the hACE2 receptor. Energy values are presented as kcal/mol

SARS2		SARS		Rs4231		Rm1		SARS2‐MUT	
K417	−93.13	V404	−2.16	V405	−1.70	V408	−1.66	K417	−98.98
N439	−0.61	R426	−127.22	N427	−0.82	A430	0.29	N439	−0.40
Y449	−8.28	Y436	−4.85	Y437	−1.53	Del.	0.00	Y449	−1.19
Y453	−1.33	Y440	−1.77	Y441	1.04	Y439	−0.69	Y453	−1.60
L455	−1.38	Y442	−4.11	W443	−1.93	S441	−1.75	L455	−1.43
F456	−3.50	L443	−1.62	V444	−0.74	Y442	−3.53	F456	−2.70
A475	−5.05	P462	−4.05	P463	−7.75	Del.	0.00	A475	−3.92
G476	−2.82	D463	58.07	G464	−3.45	Del.	0.00	G476	−1.66
F486	−3.58	L472	−3.37	P473	−2.04	Del.	0.00	F486	−5.03
N487	−6.31	N473	−4.08	N474	−1.89	N459	0.05	N487	−4.55
Y489	−3.52	Y475	−3.32	Y476	−2.75	V461	−3.52	Y489	−2.59
F490	0.30	W476	0.87	N477	0.41	Y462	−4.09	F490	0.55
L492	−1.26	L478	0.77	L479	0.25	L464	−3.24	L492	−0.78
Q493	−14.68	N479	−5.86	R480	−147.75	S465	−3.84	Q493	−18.36
Y495	−2.17	Y481	−0.73	Y482	−2.35	Y467	−4.28	Y495	−1.70
G496	−2.21	G482	−0.72	G483	−1.50	D468	105.79	D496	103.96
Q498	−4.67	Y484	−3.98	F485	−3.27	Y470	−10.31	Q498	−3.45
T500	−4.90	T486	−6.96	T487	−6.61	S472	−8.38	T500	−4.80
N501	−12.17	T487	−6.79	A488	−4.35	I473	−5.41	N501	−1.69
G502	−4.21	G488	−3.48	G489	−3.25	P474	−2.97	G502	−3.89
Y505	−5.99	Y491	−7.16	H492	−7.11	Y477	−10.14	Y505	−7.77

Strikingly, the G496D mutation (SARS2 numeration) has a large negative influence in the free energy of binding in the two complexes that contain it. It is also worth noting that the three aspartic acid substitutions present in all systems negatively contribute to the systems stability. Taking into account that the only difference between SARS2 and SARS2‐MUT is the G496D mutation, we postulate that this RBD position is critical for the human receptor recognition by coronaviruses. To the best of our knowledge, no coronavirus having aspartic acid at this position is able to infect human cells. This result supports the prediction from FUBAR analyses indicating that the site G496D is under purifying selection. Combined, our results strongly suggest that the mutation of the D496 residue present in bat SARS‐like coronaviruses is critical for their RBDs to recognize the human hACE2 receptor. Additionally, it shows the importance of sites under purifying selection in RPS for the RBD evolution.

**FIGURE 3:**
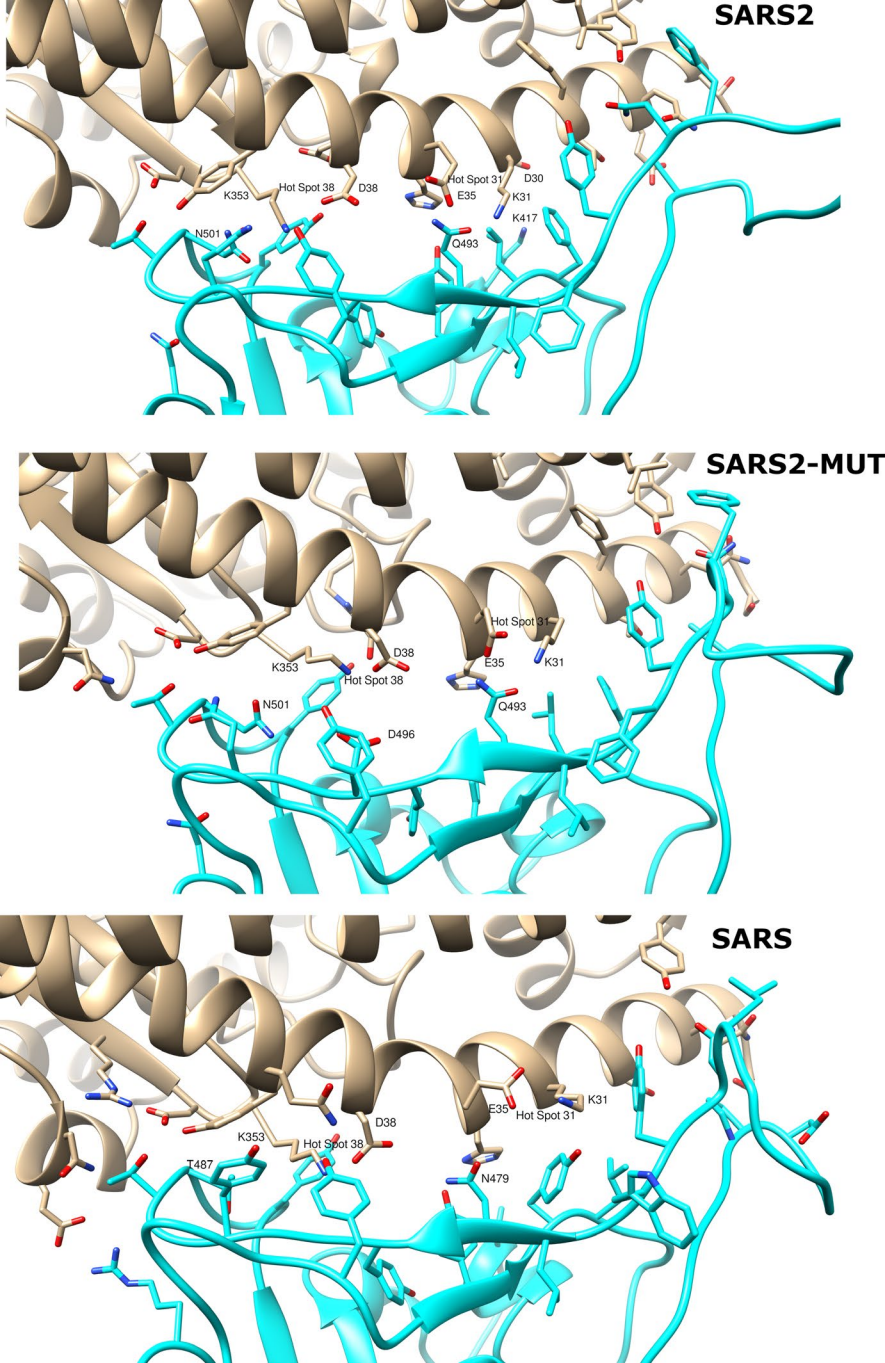
Predicted interaction of SARS2 (top), SARS2‐MUT (middle) and SARS (bottom) with the human ACE2 receptor. hACE2 in depicted in gray and the RBD of coronaviruses in cyan. Oxygen atoms are colored red and nitrogen blue. The numbering of the residues corresponds to that of the sequence of each spike glycoprotein

To better interpret the influence of the key interactions between the studied coronaviruses RBDs and the hACE2 receptor, their complexes were visually analyzed. The predicted RBD‐hACE2 complexes for SARS2, SARS, and SARS2‐MUT are depicted in Figure 3. For each complex, the structure used to create Figure 3 is the representative one of the largest cluster formed by the MD snapshots previously used in MM‐PBSA calculations.

Many studies have focused on coronaviruses mutations that favor adaptations for infecting human host infections. For example, it has been shown that specific substitutions at positions 455, 486, 4983, 494, and 501 (442, 472, 479, 480, and 487 in SARS) of the RBD of SARS favors the interaction between the RBD of SARS and hACE2 (Cui et al., 2019). Likewise, homology modeling studies found favorable interactions between the residues occupying these positions in the SARS2 RBD and the human receptor (Wan, Shang, Graham, Baric, & Li, 2020). The cornerstone of these favorable interactions is the complementarity of the RBDs with hot spots 31 and 353. These are salt bridges between K31 and E35 and between D38 and K353 of ACE2 which are buried in a hydrophobic environment (see Figure 2). In the cases of SARS2 and SARS, Q493 (N479 in SARS) and N501 (T487 in SARS) add support to the hot spots according to these previous studies (Cui et al., 2019; Wan et al., 2020). These observations should also hold for the Rs4231 strain; however, the N501A change in the later compared to SARS2 (A488 in Rs4231) add little support to hot spot 353. In this case, to continue permitting human infection, the large favorable contribution of R480 in Rs4231 to the free energy of binding could compensate the weak support provided by A488 to hot spot 353.

Interestingly, K353 is the residue forming the largest network of contacts with the analyzed RBDs among those belonging to both hot spots. Our simulations also show that in SARS2 and SARS the RBD amino acids with the largest contribution to the free energy of binding, K417 and R426 (see Table 3) respectively, do not interact with any hot spot residue. Instead, they interact with D30 of hACE2 in the SARS2 complex and with E329 of the human receptor in the SARS complex. This could indicate that interactions additional to those previously identified with the hACE2 hotspots could be critical for the stabilization of the RDB‐human receptor complexes. Finally, we analyzed the possible reasons for the predicted negative impact that the G496D mutation has on the predicted free energies of binding of the RBD to hACE2. As depicted in Figure 3, G496 directly interacts with K353 in hot spot 353 and its mutation interferes with the D38‐K353 salt bridge. Specifically, D496 of the RDB point to D38 of hACE yields a high electric repulsion between these amino acids. Consequently, this portion of the RBD is pushed to a position further from hACE2 than that observed in the wild type receptor, resulting in the reduction of its network of contacts with K353. As a result, the binding of the RBD to hACE2 is considerably inhibited and unlikely to occur.

## 4 CONCLUSIONS

A priority in ongoing research is to better understand coronavirus evolution, with specific interests in understanding the role of selection pressures in viral evolution, and clarifying how viral strains can infect novel hosts. Our experiments suggest that there are sites under positive selection in the S‐protein gene of SARS‐CoV‐2 and other betacoronaviruses, particularly in a region that we called RPS (Region under Positive Selection) inside of the RBD. However, we have identified that by in large, sites in this region (and overall, in the S‐protein gene) are under purifying selection. Particularly, for the site 496, the presence of glycine, or at least the absence of aspartic acid, seems indispensable for the interaction with the hACE2. Additionally, we performed MD simulations and free energies of binding predictions for five different complexes of betacoronaviruses that do and do not infect human cells. Our results suggest that as long as no disrupting interference occurs with both salt bridges at hot spots 31 and 353 SARS‐related coronaviruses are able to bind with hACE2. Modeling results suggest that interference with the hot spot 353 could be and effective strategy for inhibiting the recognition of the RBD of the SARS‐COV‐2 spike protein by its human host receptor ACE2 and hence prevent infections. Although additional simulations and experiments are required, all evidence suggests that the mutation of D496 in bat SARS‐like coronaviruses permit infection of human cells. Giving the large contribution of SARS2 K417 to the free energy of binding of the RBD to hACE2 we propose that blocking its interaction with the receptor D30 could be a promising strategy for future drug discovery efforts.

## CONFLICT OF INTEREST

None declared.

## Supporting information

Fig S1‐S5

## Data Availability

The phylogenetic tree presented in this manuscript was uploaded to DRYAD (https://doi.org/10.5061/dryad.w3r2280mp). The full networks of interactions between the coronaviruses and the hACE2 receptor are provided as Supporting Information.
